# Force Sensing in Surgical Sutures

**DOI:** 10.1371/journal.pone.0084466

**Published:** 2013-12-23

**Authors:** Tim Horeman, Evert-jan Meijer, Joris J. Harlaar, Johan F. Lange, John J. van den Dobbelsteen, Jenny Dankelman

**Affiliations:** 1 Department of Biomechanical Engineering, Technical University Delft, Delft, The Netherlands; 2 Department of Department of Surgery, Erasmus Medical Center, Rotterdam, The Netherlands; Toronto Western Hospital, Canada

## Abstract

The tension in a suture is an important factor in the process of wound healing. If there is too much tension in the suture, the blood flow is restricted and necrosis can occur. If the tension is too low, the incision opens up and cannot heal properly. The purpose of this paper is to describe the design and evaluation of the Stitch Force (SF) sensor and the Hook-In Force (HIF) sensor. These sensors were developed to measure the force on a tensioned suture inside a closed incision and to measure the pulling force used to close the incision. The accuracy of both sensors is high enough to determine the relation between the force in the thread of a stitch and the pulling force applied on the suture by the physician. In a pilot study, a continuous suture of 7 stitches was applied on the fascia of the abdominal wall of multiple pigs to study this relationship. The results show that the max force in the thread of the second stitch drops from 3 (SD 1.2) to 1 (SD 0.3) newton after the 4^th^ stitch was placed. During placement of the 5^th^, 6^th^ and 7^th^ stitch, the force in the 2^nd^ stitch was not influenced anymore. This study indicates that in a continuous suture the force in the thread remains constant up to more than 3 stiches away from the pulled loose end of the suture. When a force feedback tool is developed specially for suturing in surgery on patients, the proposed sensors can be used to determine safety threshold for different types of tissue and sutures.

## Introduction

Suture techniques for abdominal wound closure have been a subject of investigation for a long period of time. The incidences of post-operative complications like incisional hernia and burst abdomen are 2-20% and 1-3% respectively [1,2]. In the high risk patient, incisional hernia rates as high as 38% are found [3]. Although much is known about patient related risk factors, technical factors like suture tension have not been thoroughly investigated. In the process of wound healing, and especially the wound healing after laparotomies, the closing method plays an important role [4]. Besides the suture technique itself, the location of the incision and tension in the suture are factors that influence the quality of the healed incision [5]. Both too high and too low suture tension have a negative effect on wound healing [6-8]. Too high suture tension will lead to ischemia, edema and tissue necrosis, while too low suture tension will lead to wound dehiscence. Several studies were undertaken to determine the relation between the thread tension and the quality of the suture. In a study of Bassini et al. [9], the thread tension was measured using a metallic lamina with strain gauges. Each end of the lamina is attached to one of the wound edges with a holder device that is fixed into the tissue layer. In a study of Cummings et al. [10], a miniature deformable E shaped tensiometer with strain gauges was hooked into a suture to determine the optimal thread tension during the fixation of organs during laparoscopic procedures. The study of Klink at al. [5] shows a technique to measure the tension with a force sensing element that is placed under the knot in a single suture. During knot tying, tension is applied to the suture and force sensing element. After calibration and within some limits, the output of the force sensing element can now be related to the thread tension. Unfortunately, a simple and effective sensor method that does not influence the measured suture tension does not yet exist. Especially in case of multiple stitches in a suture, it is not clear how the force in the first stitch influences the forces applied on following stitches. The purpose of this paper is to describe the design of two separate force sensors for suture threads. The first sensor can be used to measure the force on a tensioned thread of a stitch inside a closed incision. The second force sensor is developed to hook into the thread at the loose end of a suture to measure the pulling force applied by the physician. By measuring the force applied on those two sensors simultaneously, the relation between the pulling force and the force in one of the stitches of the suture can be determined.

### 1.1 Closing the incision

The "Running" stitch is made with one continuous length of suture material used to close tissue layers which require close approximation, such as the fascia. During each stitch, the needle is driven through both wound edges and tensioned. The thread is then given to the assistant to keep the tensioned thread away from the hands of the surgeon until the surgeon finished the next stitch. Since the tensioned thread is switching hands between surgeon and assistant, a constant pulling force is difficult to maintain. 

### 1.2 General system requirements

In a previous study a maximum force of 7 N was measured on suture threads during suturing on a skin pad [11]. If a minimal safety factor of two is used, the new sensors should withstand forces up to 15 N with a working range of 0 to 10 N. Since humans can only control instruments with frequencies not exceeding 12 Hz, the sample frequency of the forces sensors and measurement system should be minimal 24 Hz. In order to investigate the relation between the pulling force on the thread and the force in the stiches placed to close the incision, an accuracy of 0.5 N was assumed to be sufficient.

### 1.3 Hook-In Force (HIF) Sensor requirements

To prevent changes in the behavior of the surgeon during the procedure, the sensor should not interfere with the hands of the surgeon. Considering the fast and dynamic actions of the surgeon during suturing, the sensor must be installed and removed easily and quickly within a maximum of 2 seconds. Installed onto the suture thread, the total weight of the force sensor should not exceed 20 gram. This corresponds with a pulling force of 0.2 N when the thread is pulled in vertical direction.

### 1.4 Stitch Force (SF) Sensor requirements

Since two parts of the incision are pressed together by the stitches of the suture, there is no part of the suture that is not in contact with the surrounding tissue. When developing a sensor that measures the force on the suture thread between the contacting wound edges of the incision, the pressure generated by the wound edges should not influence the sensing elements. However, if the influence of the tissue on the sensor cannot be prevented, it should be measurable in order to determine its impact on the sensor’s output. Since this sensor is only placed once and remains in position until the incision is entirely closed, the installation and removal time is not critical. However, to prevent too much distortion of the workflow, the maximal installation and removal time is set on maximal 20 seconds.

## Methods

### 2.1 Software

A data recording user interface for the two sensors was built in Matlab (MathWorks,Natick, MA). With this user interface (Matlab file S1 and S2), the recording can be started, stopped and an indicator can be added to the data to mark an important event in time (i.e. procedural error or unexpected event). To monitor the functionality of the sensors at any time during the measurement, two real time force vectors are plotted in the window of the user interface (Matlab file S3 and S4). If one of the sensors fails, this will be noticed. Data from both sensors is combined with a timestamp and recorded at a rate of 60Hz. The resolution was set on 0.88 millivolt per bit since a 12bit analogue digital convertor was used with an input range of 0volt - 3.6volt. The force data in arbitrary units and time data are stored in a text file. Since the relationship between the force sensor output and the applied forces in newton (N) is known after calibration, the output is computed in newton [11]. After a measurement is stopped, the user interface allows the user to analyse the recordings and to show force graphs and important parameters as Max force, Mean force, STD of the force, and Force Impulse (Matlab file S5). A zip file of the complete software package and recorded data is also available at: www.3me.tudelft.nl/index.php?id=4404, under the section supportive software and data.

### 2.2 HIF Sensor mechanical components

**Figure 1:**
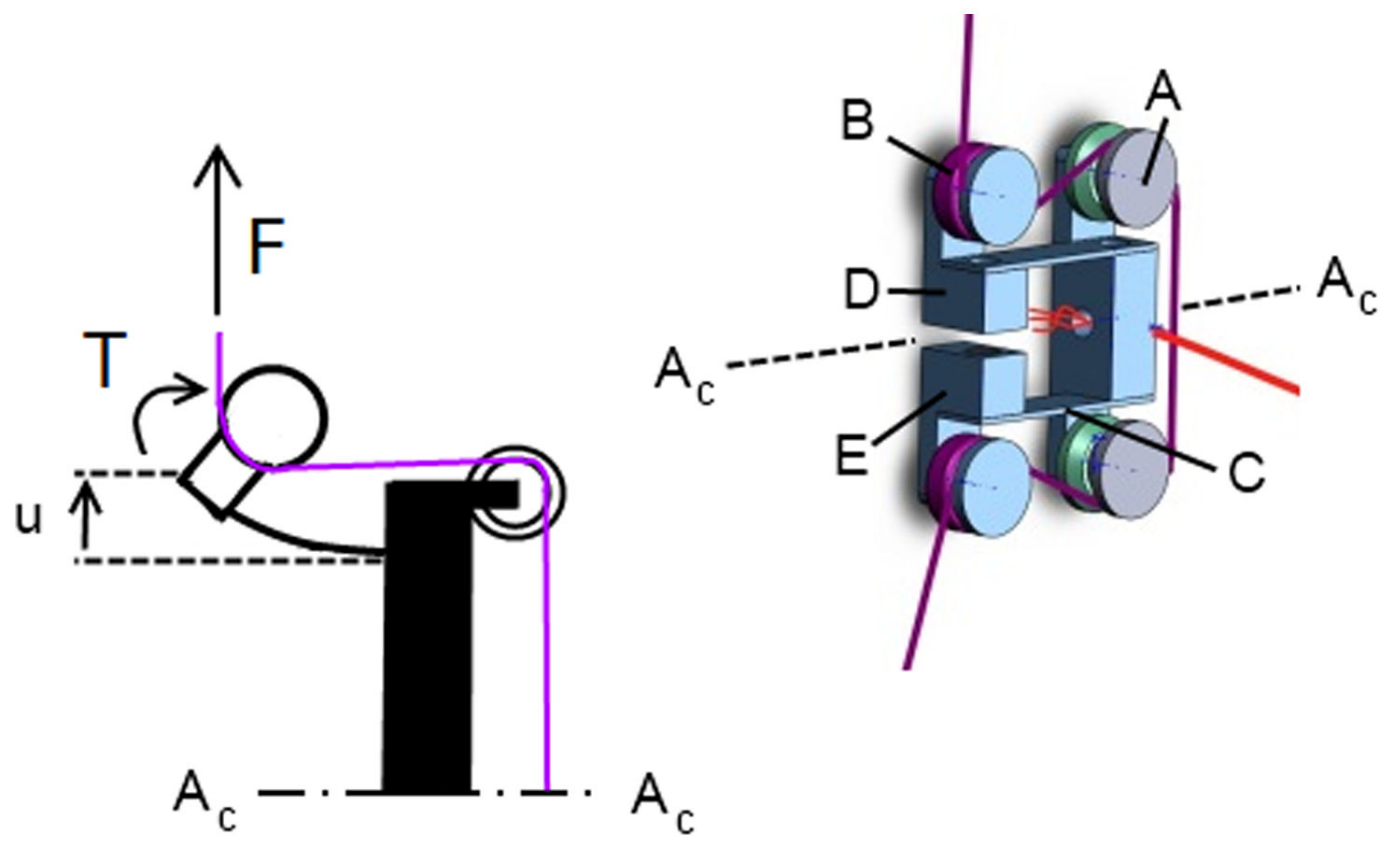
Explanation of the HIF sensor components and schematic view of the forces acting on the end of the spring blades of the HIF sensor; A-plastic discs, B- silicone discs, C-spring blade D-small hall sensor, E-magnet. Since the max. pulling force F, max. torque T and max. distance u are known, the required dimensions of the spring blade can be calculated.

A U shaped deformable force sensor with two spring blades was developed that can easily be placed onto the thread before being tensioned (Figure 1). To minimize the risk of damaging threads due to sharp edges, four discs were fabricated to guide the thread. To prevent loosening of the installed HIF sensor, the two discs at the incoming and outgoing side were equipped with an extra silicone ring. Therefore, the thread is pressed between disc and ring ensuring that the HIF sensor stays in place even when the thread is not tensioned. 

Figure 1 shows a schematic drawing of the upper side of the HIF sensor after a pulling force is applied on the thread. The force applied on the thread is counteracted by the spring blade of the HIF sensor. If the thread is loaded, the spring blade deforms (Figure 1-u) and the distance between the 2 spring blades increases.

By measuing this distance with a small inductive hall sensor attached to one spring blade (Figure 1-D) and a magnet attached to the opposite spring blade (Figure 1-E), an output in voltage is generated. The maximal displacement between magnet and hall sensor was defined as 2 mm with a minimum and maxum distance of 1 mm and 3 mm. This is the most sensitive range of the hall sensor. After calibration of the HIF sensor, the pulling force on the thread is related to the output of the hall sensor in volt.

### 2.3 SF sensor mechanical components

In comparison with the HIF sensor, the SF sensor is in continuous contact with the wound edges during the measurements. During closure, pressure is generated between the two wound edges.

**Figure 2:**
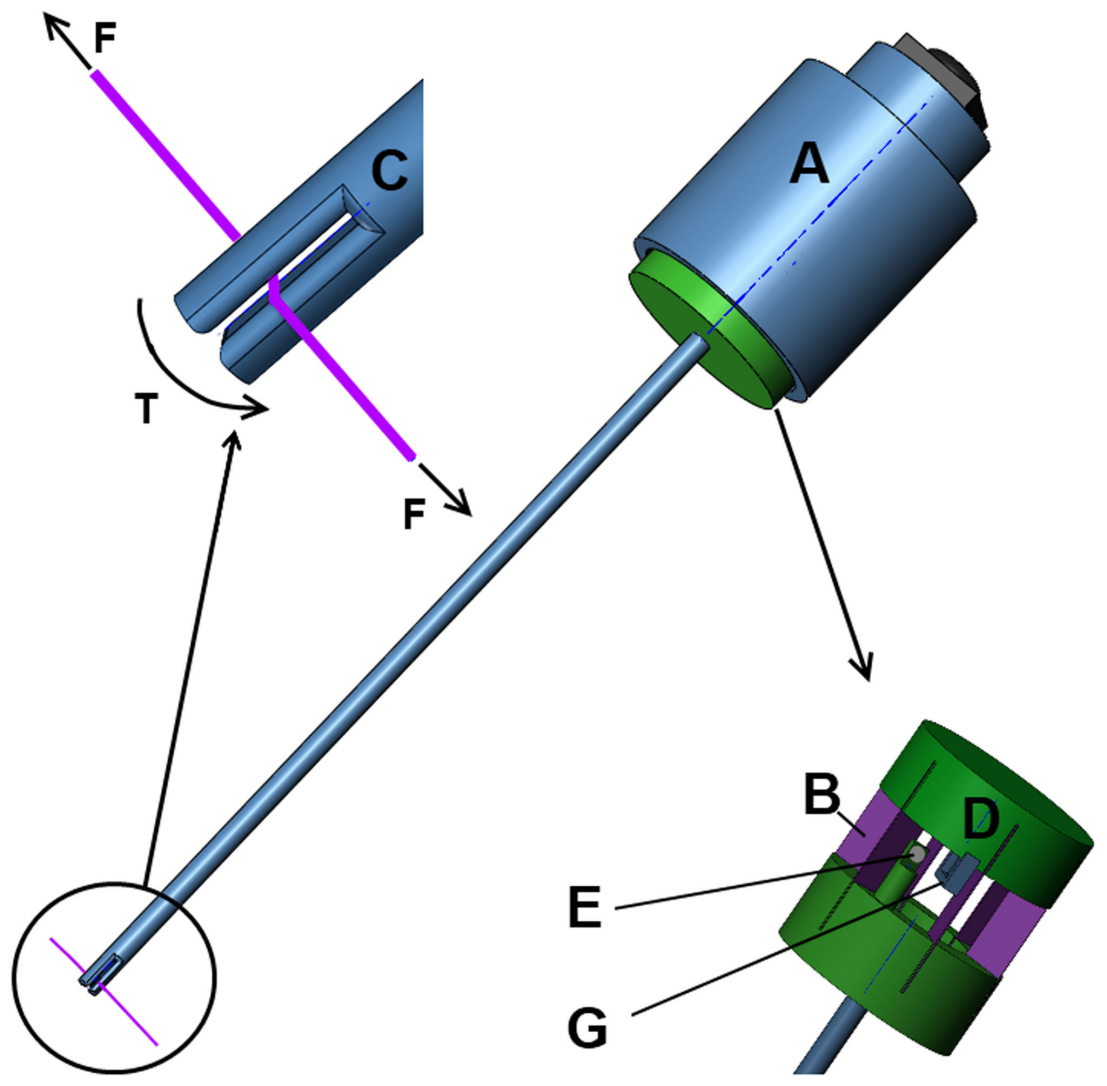
Explanation of the SF sensor components; A-housing, B- spring blades oriented in a circle, C-Close up of tip with fissure, D-hall sensor and magnet. Due to the force in the thread (F), a torque is created in the tip (T). This torque rotates the shaft in respect of the fixed housing (A). While the spring blades deform, the distance between hall sensor (G) and magnet (E) increases resulting in a change in output signal.

If a force sensor with deformable arms is installed in the incision, there is a high risk that the pressure of the wound edges on the sensor predominates the force generated by the tensioned thread. To eliminate the influence of the pressure generated by the wound edges on the sensor, a new type of force measuring concept was developed (Figure 2). Instead of measuring the deformation of an actuated arm, the tension in the thread is used to create a torque around a small round tube. In this concept, the real measurement takes place outside the abdominal wall by measuring the torque on the other side of the tube. Therefore, the required space for the measurement inside the incision is minimized to 2.5 mm and only the friction between tube and wound edges for minimal rotations of the tube need to be considered. 

The tip of the tube has a small fissure in the middle in order to place the tip over the thread (Figure 2-C). After placement, the sensor is rotated 90 degrees or more before the thread is tensioned. Since the diameter of the tube is constant, the measured forces can be calculated directly after calibration of the sensor. Only if the thread is overlapping after multiple turns, the radius is changing and the output cannot be trusted anymore. If some attention is paid during placement of the tip around the sensor this can be prevented easily.After calibration of the SF sensor, the pulling force on the thread is related to the output of the hall sensor in volt.

### 2.4 Calibration

The sensors were calibrated separately with standardized weights of 50, 100, 250, and 500 gram that where placed on a weight holder with hook. A vertically stretched thread was guided through the tip of the SF sensor and connected to the hook of the weight holder. A camera holder was modified to keep the SF sensor in place. Since there was no need for pulley’s to load the sensor, the forces are well defined and not influenced by friction in the setup. The same setup was used to calibrate the HIF sensor. In this setup, the SF sensor was removed and the hooking sensor was installed onto the thread. During the force calibration, the load on the sensors was increased from 0 to 1000 gram in steps of 100 gram. Each sensor was calibrated three times. After calibration, regression lines were added to the sensor output data to determine the relation between output and force on the thread in newton. The relation between stitch force and sensor output is of a higher order due to a higher order dependency between magnet and hall sensor distance and hall sensor output. Therefore, a second order polynomial was used for the calibration (Rout Square >0.99).

### 2.5 Accuracy

**Figure 3:**
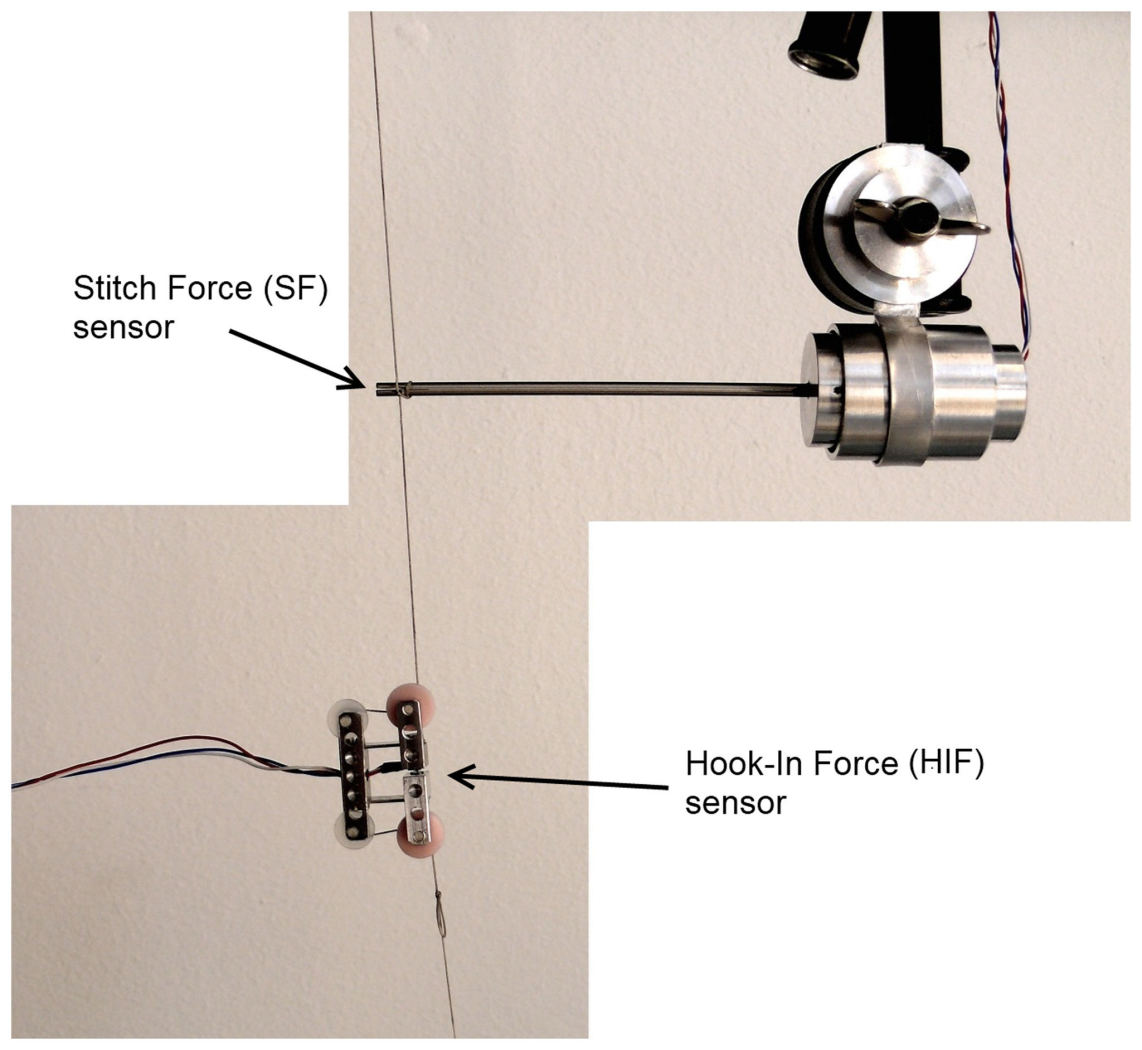
Accuracy test setup. Both sensors are installed in one thread. The left side of the thread is fixated while tension is applied on the other side of the thread with a calibrated spring balance.

To test the accuracy, both sensors were installed onto a vertically tensioned thread as shown in Figure 3. By comparing the force-time curves in one plot, differences in force output can be determined. The thread was loaded with 100 gram, 200 gram, 300 gram and 550 gram.

Although the rotation of the bar under loading of the HIF sensor is small, it is possible that the wound edges influence the measurement with the SF sensor after sticking to the metal pin while rotating. To determine this influence, the tip of the SF sensor was compressed between two 25 cm^2^ square pieces of abdominal wall to mimic the wound edge pressure (Figure S1) The pressure was set on 2 N/mm^2^ and 2.8 N/mm^2^ to mimic an extreme wound edge pressure that normally is not expected in practice. After pressure was applied we rotated the shaft from 0 to 10 degrees (i.e. two times the expected rotation of the SF sensor under maximum thread tension) for three times to record the reaction force resulted from stick-slip effects and friction. If the measured force remains below 0.5 N we consider the influence of stick-slip and friction in our studies negligible.

### 2.6 Experimental validation – setup

**Figure 4:**
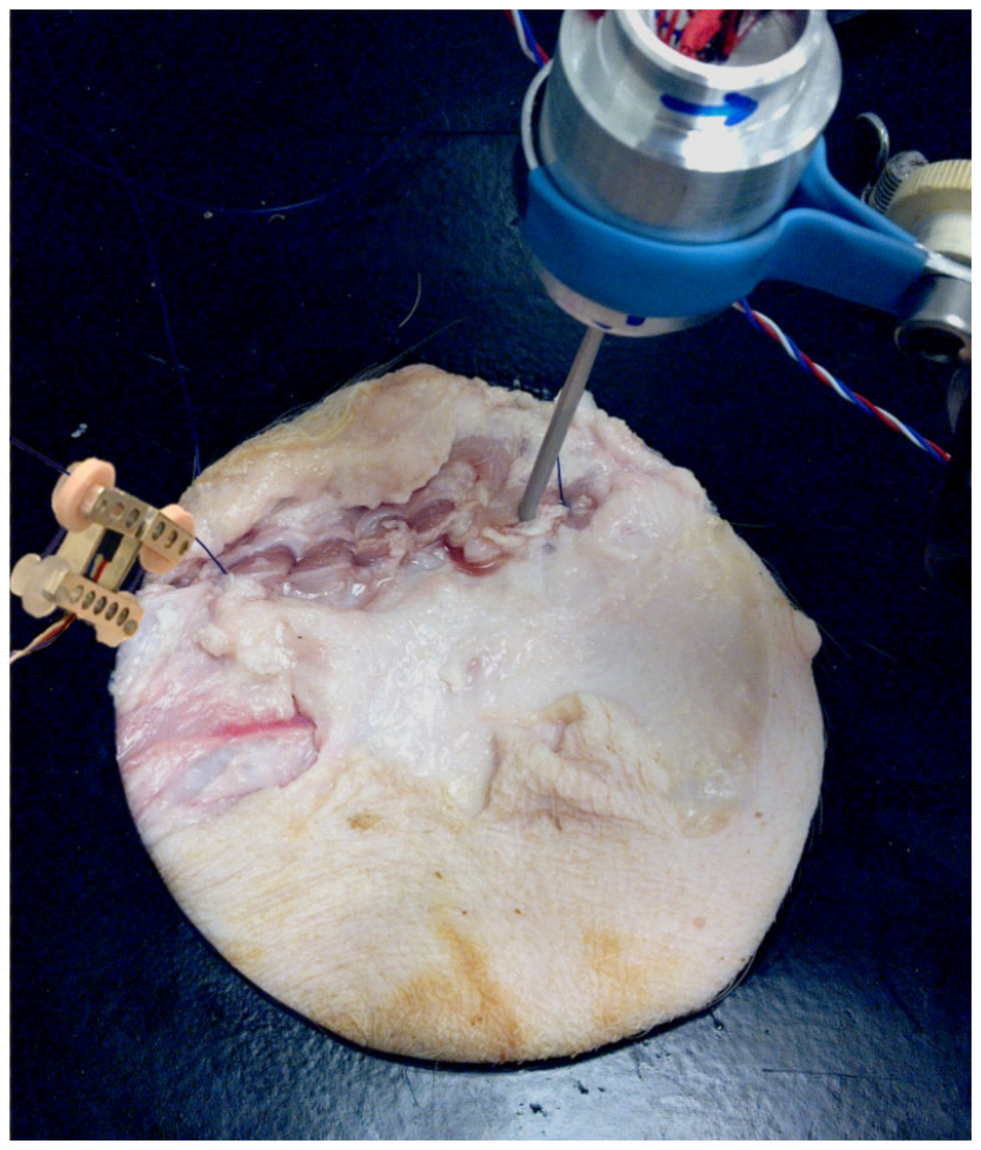
experimental validation setup after placement of the last stitch. the SF sensor is installed at the right side of the incision while the HIF sensor is installed on the pulled thread left of the incision.

Three different square porcine abdominal wall specimens of 300 by 300 mm were used during the experiments. The butcher (Keurslager J. Hoogeveen, Voorschoten, The Netherlands) prepared the samples under supervision of the leading author and gave permission to use these specimen for research. After the abdominal wall was collected from the porcine, they were frozen immediately until the experiments started. The defrosted abdominal walls were clamped between two plates for perfect fixation. During installation of the abdominal wall, sutures on each of the four corners of the abdominal wall were used to stretch the abdominal wall before the plates were pressed together. A hole was cut in the front with a diameter of 200 mm in order to make the incision and to apply the sutures. The incision was 80 mm long and a continuous suture with 7 stitches was used for closure (Figure 4). Each abdominal wall was used for two closures. After the first closure, the suture thread was removed and the procedure was repeated for a second time. During the second attempt, the needle was inserted in an undamaged part of the fascia.

### 2.7 Experimental validation – procedure

**Figure 5:**
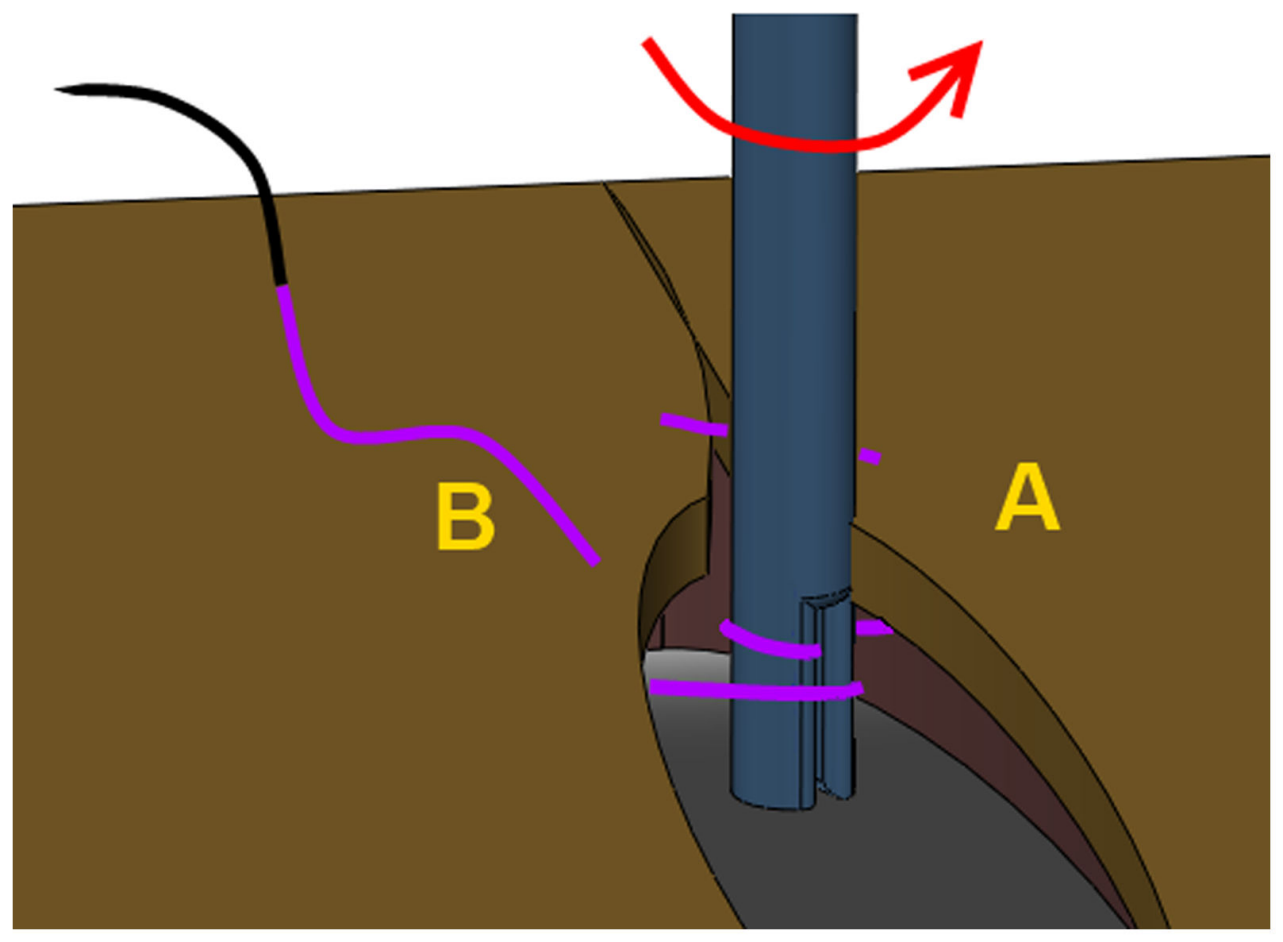
Installation of the SF sensor. The tip is placed over the thread and rotated towards point A until half of the tip is in contact with the tissue.

The closure procedure started with a knot in the first stitch. After the needle was driven through both wound edges during the second stitch, the tube of the SF sensor was placed over the exposed thread between the wound edges. Figure 5 shows the next step. The sensor is rotated until the tip touches the wound edge at side A. At the moment the tip was rotated half into the wound edge, the SF sensor was fixed inside the holder and the suture was continued until an additional 6 stitches were made. A hinge between holder and sensor allows free movement of the tip parallel to the thread. Therefore, small movements of the incision due to pulling forces on the thread do not result in a reaction force on the tip. This ensures that only the force in the thread is measured. 

## Results

Completely assembled but without threads, the mass of the HIF sensor is 16.6 gram and the assembled SF sensor weights without thread 54 grams. The maximum allowable force on the suture thread is 20 N before it damages inside the sensors. The maximum allowable working range of the SF sensor is 0 - 15 N in order to minimize tip rotation after loading and to maintain accuracy. The maximum allowable working range of the HIF sensor is 0 - 20 N before spring blades start to deform permanently. Since the expected normal working forces are much lower the SF and HIF sensor are calibrated with a maximum force of 10 N. In a conventional workshop the production of each sensor used in this study (not optimised for large scale production) took approximately six hours and an additional two hours was required for calibration of the set.

### 3.1 Calibration

**Figure 6:**
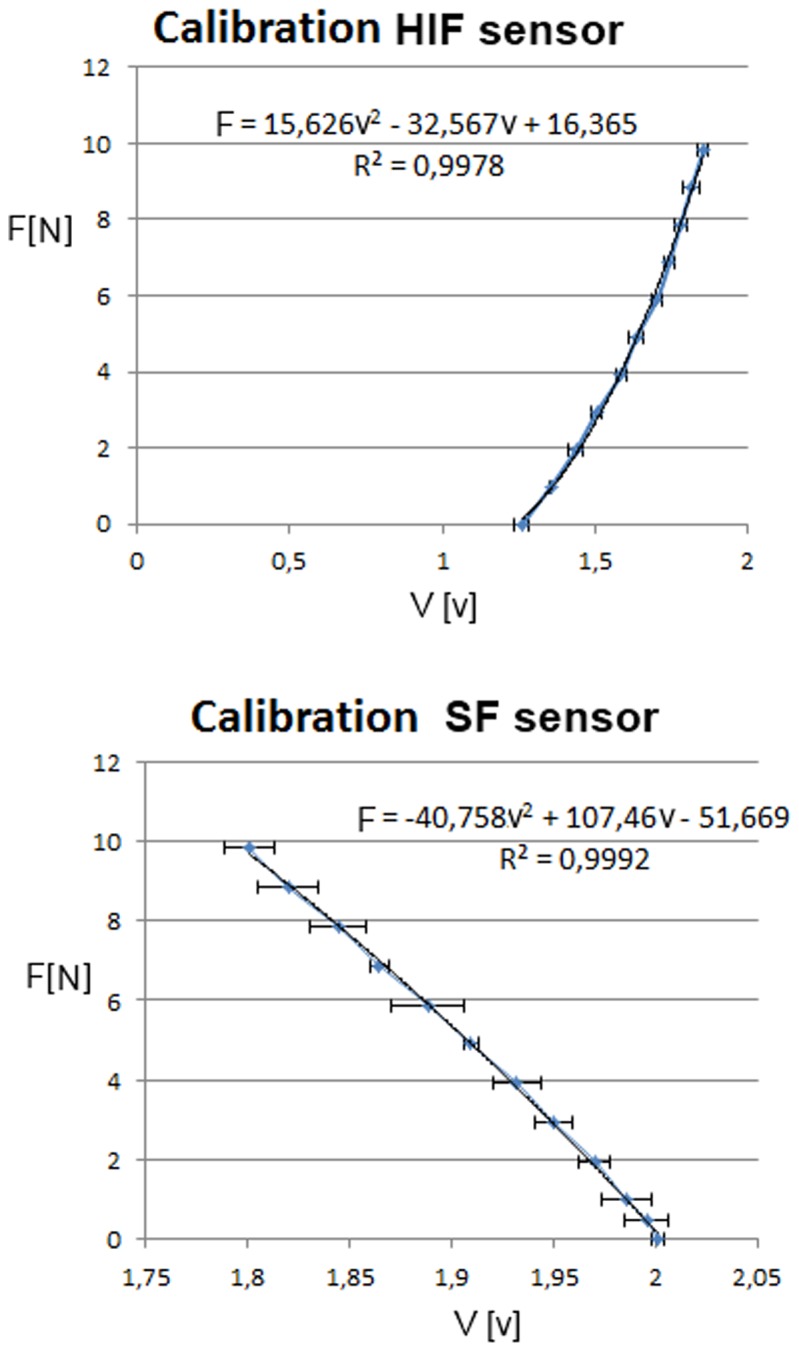
Calibration graphs of Hook-In with regression lines, R^2^ fit and 95% CI. Each data point represents the average of 3 measurements per load value.

Figure 6 presents the regression lines and Rout Square values for the averaged data of each sensor. In both cases a 2^nd^ order polynomial seems sufficient for accurate calculation of the force from the sensor output.

### 3.2 Accuracy

**Figure 7:**
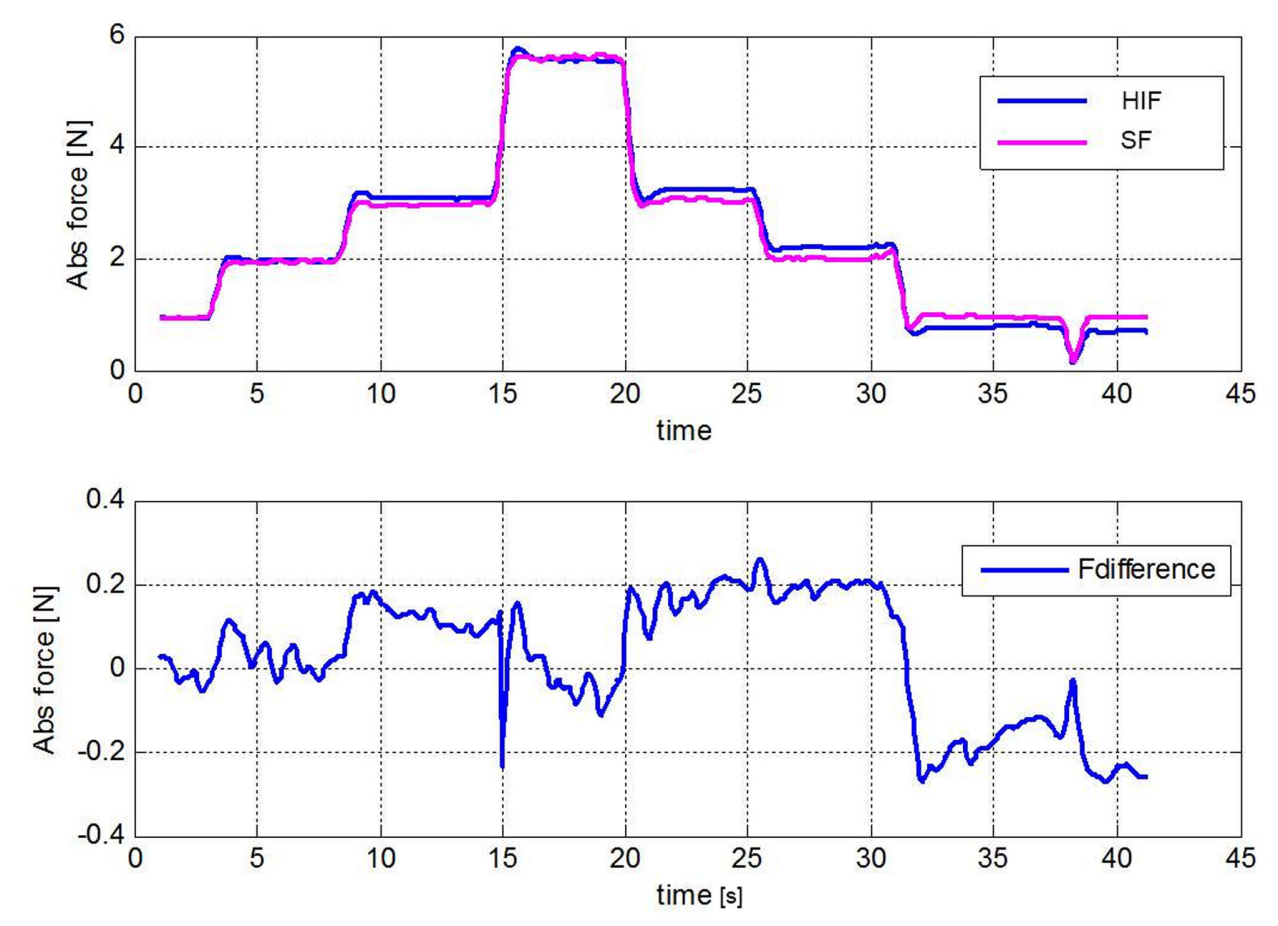
Accuracy of the sensors. Upper graph; the output in newton from the HIF sensor and SF sensor during one loading cycle. Lower graph; Fdifference indicates the difference between HIF and SF output in newton.

The measurements indicate that both sensors can easily detect force differences of 0.05 N. The upper graph of Figure 7 shows the force graph of the SF and HIF sensor that both measure the force in a single thread (Figure 3 for setup). The lower graph shows the difference in output of the sensor during the complete loading cycle. An average error of 0.025 N is found for the complete measurement. The measurements (Figure S2) performed to determine the influence of the pressurized abdominal tissue on the rotating tip showed a low reaction force at even the highest pressure (Max. 0.22 N at 2.8 N/mm^2^). 

### 3.4 Experimental validation

In real practice it was possible for the surgeon to install the SF sensor within 20 seconds and to remove it within 2 seconds. The HIF sensor can be installed within two seconds after some practice. Placement is easiest if the sensor is held at the aluminum base with the preferred hand and the thread is guided around the four discs with the other hand. Removal from the thread of the HIF sensor took less than one second in all 6 trials. Figure 4 shows the setup at the end of the suture.

**Figure 8:**
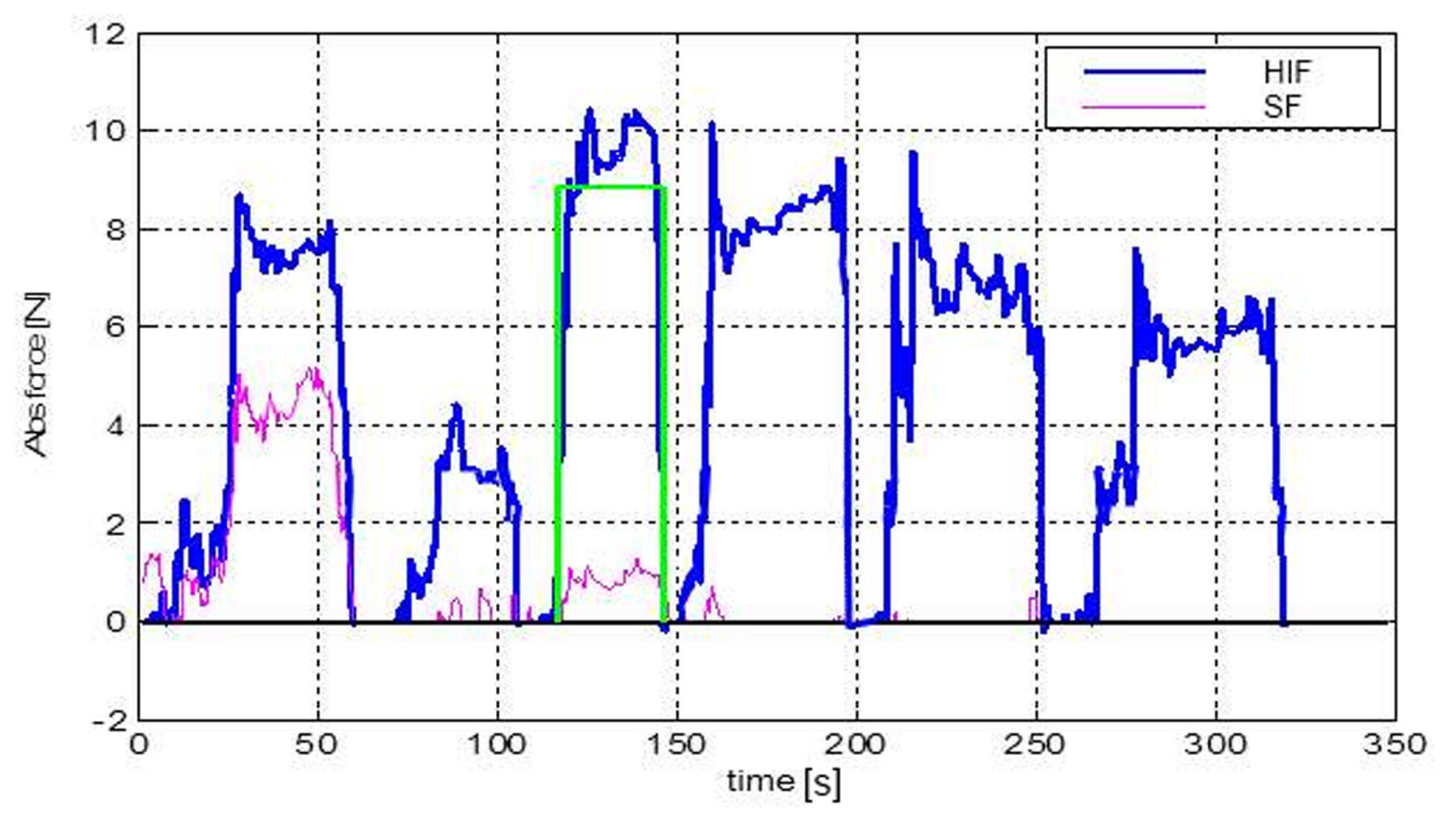
Force graphs from the HIF and SF sensors for a continuous suture with 6 stitches. SF output; force in the thread of the second stitch. HIF output; force in the thread 50 mm under the needle. Rectangle; indicator that shows where the highest mean force in the HIF sensor was found.

**Figure 9:**
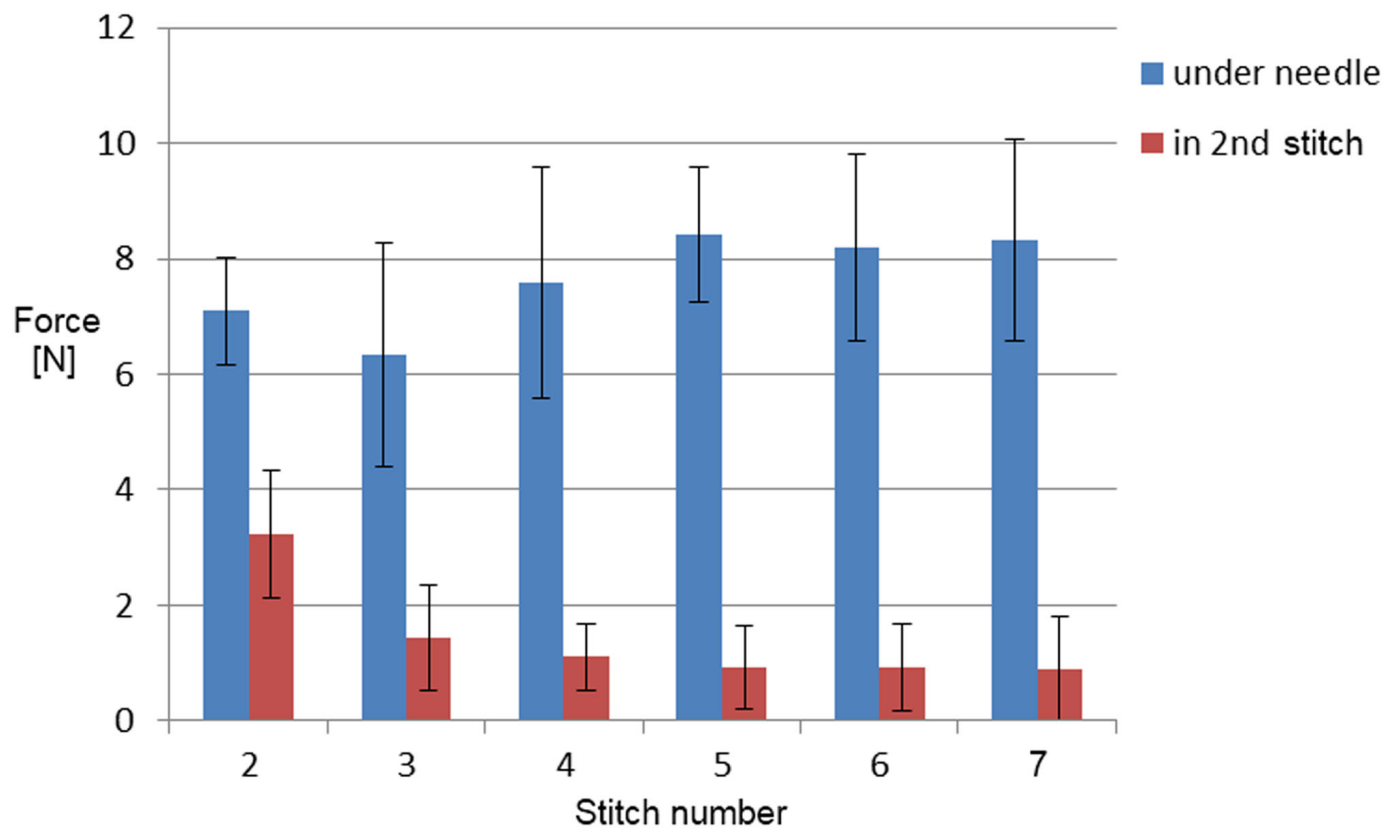
Force graph of averaged force per stitch with SD of all six measurements. The force in the 2^nd^ stitch was measured with the SF sensor and the force in the thread 50 mm under the needle was measured with the HIF sensor. Stitches were placed in the fascia.

Figure 8 shows a plot of the forces acting in the second stitch in the incision (measured by SF) and in the thread 50 mm under the needle (measured by HIF). Figure 9 shows that the force in the thread of the 2^nd^ stitch become constant after the 4^th^ and following stitches are placed. 

## 4 Discussion

In this study two new sensors (SF and HIF) for measuring the forces on sutures were designed, produced and evaluated. Experiments showed that the sensors are robust and accurate enough to measure the pulling and stitch force during suturing and that stick-slip effects and friction between SF tip and wound edges can be neglected.

### 4.1 Experimental validation

We found that due to a relatively high resistance of the tissue in every stitch during placement of the 5th, 6th and 7th stitch, the force in the 2nd stitch was not influenced anymore. This means that when sutures are not pulled through properly after each stitch there will be an imbalance of the divided forces in the wound. The suture with the highest tension on the fascia is most vulnerable for a cut through the fascia or development of necrosis. This means that every stitch should be pulled through with the same strength to lower the risk of wound failure. The remaining thread tension in the second stitch (1.0 N SD 0.6) is in the same force range as the loop tension found in a single stitch placed in the skin and muscle layers after 6 minutes in the study of Klink et al (1.2 N SD 0.5) [5].

### 4.2 The value of force information

With the proposed HIF and SF sensor concepts, objective comparison becomes possible between different types of surgical sutures and suture techniques. Safety thresholds for thread tension can now be determined for different types of tissues. This information can be used for surgical training systems that inform the trainee about risks related to tissue tear during suturing [11-13]. Furthermore, the suturing process can be optimized if the forces acting in the threads are known at all time.

### 4.3 Towards a practical feedback tool

Extra usability test performed with two surgeons, two residents and two researchers indicated that all test subjects without prior knowledge about the sensors were able to install the SF sensor in 7.8 seconds (SD 7.1) and the HIF sensor in 10.2 seconds (SD 7.5) on a mock-up of the experiment (Table S1). Moreover, the validation study showed that the installation time can be reduced to a couple of seconds. Therefore this combination of sensors proved useful to determine the relation between the force in the thread of a stitch and the pulling force.

**Figure 10:**
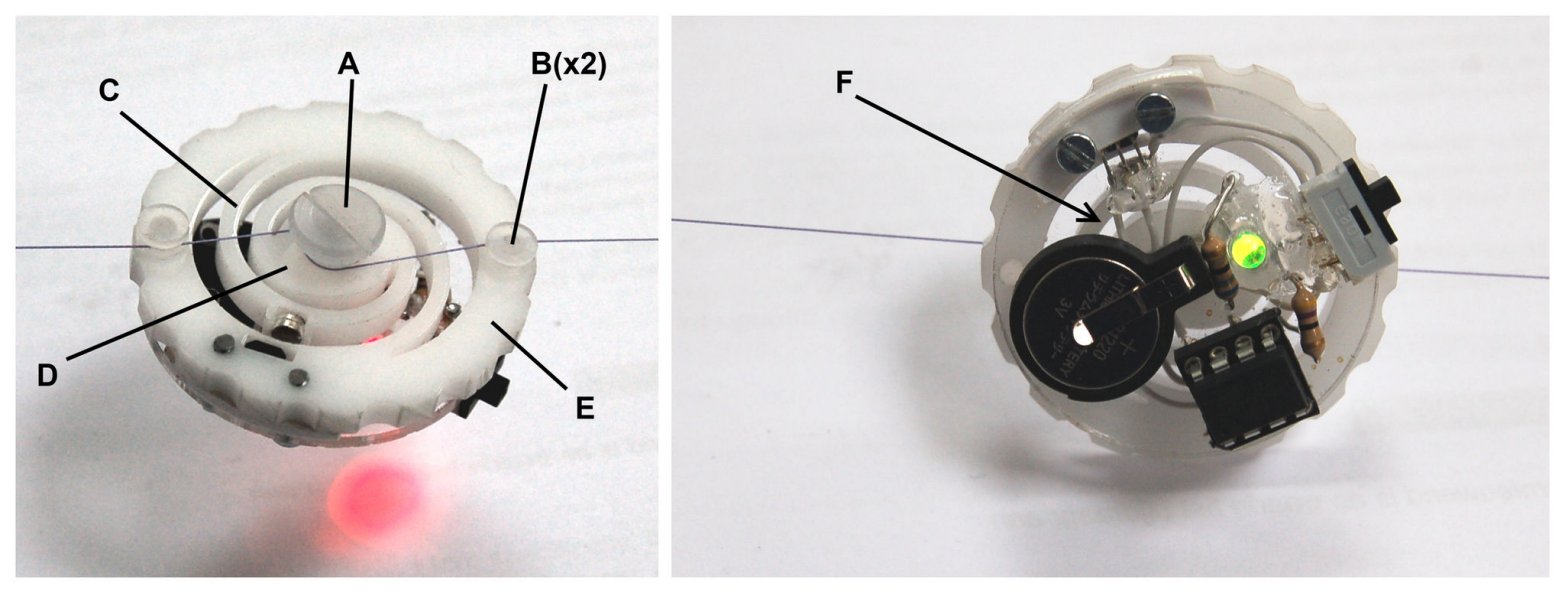
A simple “wheel” sensor with embedded measurement and feedback system. Left, force exceeds 10 Newton and LED turns red. Right, force is between 8 and 10 Newton and LED turns green. A-Inner pin, B-external pins, C-spiral shaped bar, D-inner ring, E-external ring, F-embedded electronics for force feedback.

Although the SF and HIF sensor are useful for research purposes, for training during surgery in the OR, a simple small, light and affordable sensory system with a simple interface is preferable to inform the user about the magnitude of the pulling force. Therefore a new “wheel ” sensor was developed that can be laser cut from any suitable relatively stiff medical grade plastic and three machined pins that are fixed in three holes in the wheel. This wheel sensor supports itself between the tensioned threads and is easy to install and remove (Figure 10-A,B). Comparable to the SF sensor placement in this study, the fissure in the inner pin of the wheel sensor is placed over the tensioned thread before the wheel is rotated 180 degrees. After rotation, the outer pins are hooked behind the thread. After loading, the inner pin rotates in respect to the two external pins and the spiral shaped bars (C) that connect the inner (D) and external (E) ring of the wheel are pressed outwards. Since the external ring contains the hall effect sensor and the spiral shaped bar the magnet, the pulling force can be related to the output signal of the hall sensor after calibration. 

When the control system, power source and feedback source are small enough they can be embedded in the sensor itself. Figure 10 shows a prototype of the wheel sensor with embedded feedback system. The system is controlled by an ATtiny85 micro controller that operates at 100hz and powered by a 3v Lithium battery. The complete prototype of Figure 10 has a mass of 11.3 gram. If smd technology with a custom circuit board is used it is estimated that the weight can be reduced to 8 gram. If this feedback sensor is used during training, a green LED indicates a safe working range for the pulling force (Figure 10-Right). If the multi-colour LED on the sensor turns red, the sensor warns the surgeon that the pulling force exceeds a predefined threshold (Figure 10-Left). In a later phase of development, usability tests and a cost prize calculation should indicate if it is feasible to put this disposable pulling force sensor on the market.

### 4.4 Limitations of this study

We performed 6 series of stitches on three different specimen. Based on visual inspection we chose parts of the prepared fascia that was undamaged to insert the stitch, therefore it is unlikely the first suture placement influences the force measured during the second suture. We recorded the data from the first point of insertion of the needle till the last knot was made before we took the suture out or replaced the specimen. In our study we did not measure the force in the stitch over time. The results of Klink et al. showed a drop of loop tension in single stitches in skin and muscle layers of a rodent model after 60 minutes. Hence it is difficult to estimate whether the drop of the stitch force in the second stitch of Figure 9 is caused by a decreasing influence of the pulling force or that tissue relaxation also reduced the stitch force. Therefore, further studies are required to investigate the role of tissue relaxation in continued sutures.

## Conclusion

A measurement system is developed that can be used to measure forces in suture threads inside and outside the incision. With the presented force measurement system it becomes possible to relate the thread tension inside sutures to the pulling force applied by the physician. Therefore it enables the comparison of different suture techniques and to determine their impact on wound healing giving insight in one of the oldest surgical procedures. This can lead to a simple hand tool that warns surgeons about excessive forces on suture threads and thereby reduce postoperative complications like incisional hernia and burst abdominal wall.

## Supporting Information

Figure S1 **Test setup of the Stick-slip and friction measurements.**
(TIF)

Figure S2 **Results of the Stick-slip and friction measurements.**
(TIF)

Table S1 **Installation time of SF and HIF sensors.**
(XLSX)

Matlab File S1 **User interface Figure file.**
(FIG)

Matlab File S2 **User interface Matlab file.**
(M) Click here for additional data file.

Matlab File S3 **Part of the software that records the force data in a loop.**
(M) Click here for additional data file.

Matlab File S4 **Part of the software where the force arrow is generated that is visualised in the user interface during recording.**
(M) Click here for additional data file.

Matlab File S5 **Part of the software that analyses the recorded data to display the parameter results in the user interface.**
(M) Click here for additional data file.

## References

[1] Mudge M, Hughes LE (1985) Incisional hernia: a 10 year prospective study of incidence and attitudes. Br J Surg 1: 70-71. PubMed: 3155634. 10.1002/bjs.1800720127 3155634

[2] Webster C, Neumayer L, Smout R, Horn S, Daley J, Henderson W et al. (2003) Prognostic models of abdominal wound dehiscence after laparotomy. J Surg Res 2: 130-137. PubMed: 12643854. 10.1016/s0022-4804(02)00097-5 12643854

[3] Bevis PM, Windhaber RA, Lear PA, Poskitt KR, Earnshaw JJ et al. (2010) Randomized clinical trial of mesh versus sutured wound closure after open abominal aoartic aneurysm surgery. Br J Surg 10: 1497-1502. 10.1002/bjs.7137 20603858

[4] Rath A (2008) The healing of laparotomies: review of the literature. Hernia. 2: 145-149.

[5] Klink CD, Binnebosel M, Alizai HP, Lamberts A, Vonstrotha KT et al. (2011) Tension of knotted surgical sutures shows tissue specific rapid loss in a rodent model. BMC Surgery. 11(1): 36. doi:10.1186/1471-2482-11-36. 22188826 PMC3275509

[6] Högström H , Haglund U, Zederfeldt B (1990) Tension leads to increased neutrophil accumulation and decreased laparotomy wound strength. Surgery, 107(2): 215-219. PubMed: 2154055. 2154055

[7] Höer J, Klinge U, Schachtrupp A, Töns C, Schumpelick V (2001) Influence of suture technique on laparotomy wound healing: an experimental study in the rat. Langenbecks Arch Surg, 386(3): 218-223. doi:10.1007/s004230000196. PubMed: 11382325. 11382325

[8] Stone IK, Von Fraunhofer JA, Masterson BJ (1986) The biomechanical effects of tight suture closure upon fascia. Surg Gynecol Obstet, 163(5): 448-452. PubMed: 3535137. 3535137

[9] Bassini R (1988) Method of measuring suture tension in surgery. Medical and Biological Engineering and Computing, 26: 451-454. 3076603 10.1007/BF02442310

[10] Cummings JF (2000) A miniature suture tensiometer for laparoscopic applications. J Invest Surg, 13(5): 253-258. doi:10.1080/08941930050206265. PubMed: 11071560. 11071560

[11] Horeman T, Blikkendaal MD, Feng; Dijke A D van, Jansen FW et al. (2013) Visual Force Feedback Improves Knot-Tying Security. Journal of Surgical Education. 10.1016/j.jsurg.2013.06.021 24411436

[12] Horeman T, Rodrigues SP, van den Dobbelsteen JJ, Jansen FW, Dankelman J (2012) Visual force feedback in laparoscopic training. Surg Endosc, 26(1): 242-248. PubMed: 21858573. 21858573 10.1007/s00464-011-1861-4 PMC3242944

[13] Oshima N, Aizudding M, Midorikawa R, Solis J, Ogura Y et al. (2007) Development of a Suture/Ligature Training System designed to provide quantitative information of the learning progress of trainees Robotics and Automation, 2007 IEEE International Conference on (10)14. pp. 2285-2291.

