# Supplementary figures and images for: Force Sensing in Surgical Sutures
Source: PLoS One. 2013 Dec 23;8(12):e84466. doi: 10.1371/journal.pone.0084466 (PMC3871579; doi:10.1371/journal.pone.0084466)

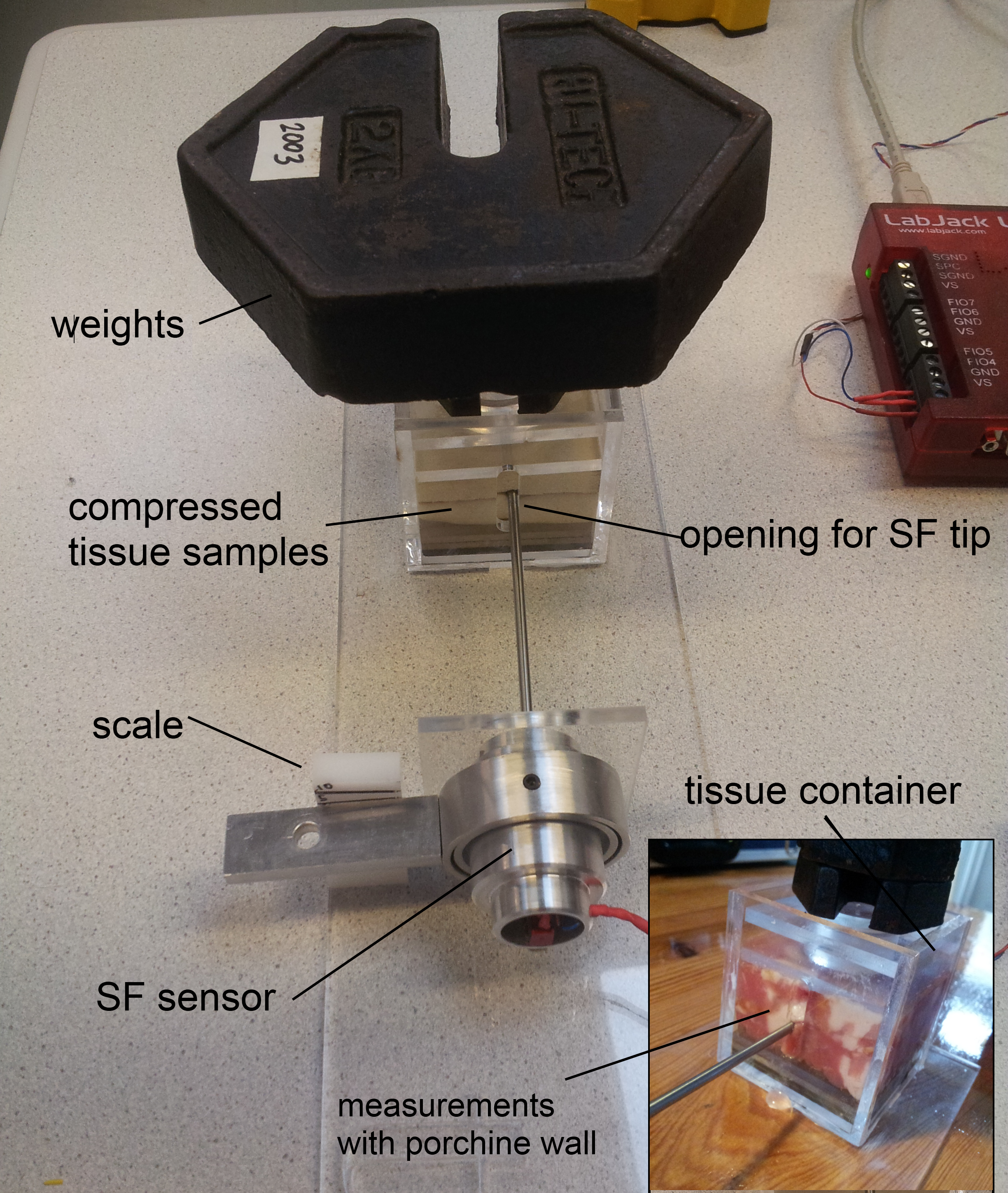

Supplement: Figure S1 — Test setup of the Stick-slip and friction measurements. (TIF) [file pone.0084466.s001.tif]

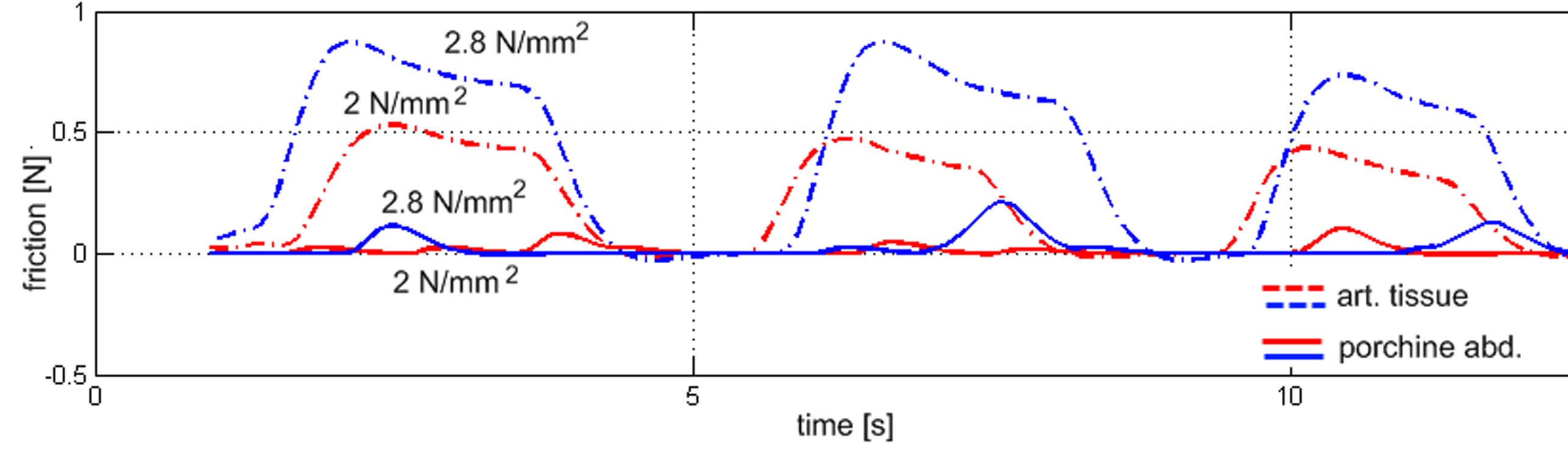

Supplement: Figure S2 — Results of the Stick-slip and friction measurements. (TIF) [file pone.0084466.s002.tif]
